# Microbial Musings – November 2021

**DOI:** 10.1099/mic.0.001134

**Published:** 2022-01-12

**Authors:** Gavin H. Thomas

**Affiliations:** ^1^​ Department of Biology, University of York, York, YO10 5YW, UK

As we approach the end of the year, another new variant of the severe acute respiratory syndrome coronavirus 2 (SARS-CoV-2) has emerged and is quickly spreading around the world. The variant is sufficiently different to justify a new letter in the naming scheme of the World Health Organization (WHO). While early indications of its increased infectivity appear to be bearing out, it will hopefully not be linked to increased disease severity or strong vaccine evasion, but we may be in for another tough winter. The new found familiarity of the word omicron, the written form of a Greek letter that is not widely used by microbiologists, will at least help me improve my pronunciation of the human gut bacterium *Bacteroides thetaiotaomicron *, which, as I mused last July, takes its names from exhibiting morphologies that resemble the shapes of theta, iota and omicron [1]. Looking at my Greek alphabet, as one does, I hadn’t noticed before that various letters have been skipped, as the WHO didn’t want the names to cause confusion or cultural offence, so they skipped past Nu because it sounds too close to ‘new’ and Xi because it is a common surname, hence omicron takes the name for this new variant and will be imprinted on the public’s collective memory for the next generation.

We start this month with two papers from the Antimicrobials and AMR section of the journal. The first is from the group of Fernando Luciano at the Pontificia Universidade Catolica do Parana, Brazil [2]. This is an interesting paper that tests a potentially cheap approach to treat animal feed to reduce carriage of *Salmonella* in livestock. The approach is to prepare cell-free supernatant from other bacteria that are known to have antagonistic effects on *Salmonella enterica* [3]. These were prepared from a range of lactobacilli and they find that many of the extracts are active, although the effects appears to require low pH and are lost when they are neutralized. This is consistent with their hypothesis that small natural products are responsible for killing as bacteriocins produced by *Lactobacillus rhamnosus* that are active against *S*. Typhimurium function best at pH 4.5 [4]. The lyphophilized extracts are also able to function to reduce *Salmonella* in a simulated swine digestion model and while this is still very preliminary, it nicely demonstrates the principle of a cheap alternative to traditional antibiotics using crude extracts.

The second paper concerns the mechanism of the antibiotic colistin (polymyxin E). Just over 10 years ago I remember hearing from David Livermore, a leading expert on antimicrobial resistance (AMR) in the UK, at a Royal Society of Chemistry meeting on AMR. He talked about the rising levels of resistance to β-lactams despite the impressive efforts of new derivatives being made – the ‘pharma composes, bacteria disposes’ cycle! When discussing a patient with resistance to multiple β-lactams, he mentioned that it could be treated by an antibiotic, colistin, that I hadn’t heard of before [5]. Colistin had been removed for many years from clinical use and was a ‘back of the medicine cabinet’ antibiotic. Looking back at my go-to undergraduate textbook on antimicrobials by Franklin and Snow [6], colistin is not even mentioned and the polymixins only in passing due to having “a minor place in medicine because they also damage mammalian cell membranes”. However, significant increases in AMR have led to this antibiotic being increasingly called upon [7]. The lack of interest in colistin until quite recently perhaps explains why a complete understanding of its mechanism of action has been lacking. With many new antibiotics a quick way to identify the mechanism of action is through the isolation of resistant mutants that alter the target site, for example in the recent discovery of darobactin and the identification of BamA as its target [8]. For colistin resistance there are a number of well-studied routes, which normally involve the modification of the lipopolysaccharide (LPS) to increase its positive charge [9]. This was thought to then reduce the binding of the positively charged antibiotic at the outer membrane (OM), reducing the ability of the antibiotic to reach its final target of the cytoplasmic membrane (CM). While polymixins have been known to be active at this location for many years [10], the precise mechanism of action was unknown. To explain the exciting new paper in this issue from the group of Andrew Edwards (@bugsinblood), it is worthwhile explaining the group’s important paper published earlier this year, where they first found new evidence of how colistin kills bacteria [11]. This work led by Akshay Sabnis from Edward’s group used a combination of elegant methods, combined with examining how resistance mediated by the MCR-1 protein is mediated. MCR-1 is a phosphoethanolamine (pEtN) transferase that modifies the LPS core to increase its positive charge and repulse the cationic colistin molecule [9]. However, in this paper they showed that this modification did not protect the OM from disruption by colistin. Given that the LPS is assembled in the cytoplasmic membrane before its export to the OM, they then examined if the altered LPS in the inner membrane was preventing disruption of CM function and indeed this is what they observed [11]. Consequently, they proposed that colistin is targeting LPS when it is still in the CM and to support this they used another antibiotic, murepavadin, that triggered accumulation of LPS in the CM and found that the cells had increased sensitivity to colistin. In their latest paper in this issue of *Microbiology* they extend this study by examining whether the same phenomenon is consistent with other mechanisms of colistin resistance, using a range of other clinically relevant plasmid-based *mcr* genes and *Escherichia coli* strains with chromosomal mutations. The work, led by Madeline Humphreys, alongside Gerald Larrouy-Maumus (@gLarrouyM_Lab), Christopher Furniss (@furnissrc) and *Microbiology* editor Despoina Mavridou (@bondSSbond), with Edwards at Imperial College London, UK, used methods that could selectively disrupt either the OM or the CM. They assessed the ability of resistance genes that conferred the same modification or in combination with the alternative 4-amino-4-deoxy-l-arabinose (l-Ara4n) modification or both together. Realizing that the disruption of the OM by colistin could have other useful clinical outcomes, they show that treatment with rifampicin, which normally has a high minimum inhibitory concentration (MIC) against *E. coli *, in the presence of sublethal concentrations of colisitin, now sensitizes the bacteria to this cytoplasmically active antibiotic. This is consistent with some recent work from the Brian Coombes (@BrianKCoombes) and Eric Brown labs (@eric_brown_bbs) at McMaster, University, Canada [12], and Tim Walsh’s work with Ya-Hong Liu’s group at the South China Agricultural University [13], and could well have highly useful clinical applications.

Our next paper relates to metals and microbes and concerns an important family of proteins that have one of the best understood functions in intracellular metal storage in bacteria. These are the ferritin proteins, which form large 24 mer rhombic dodecahedral structures, not dissimilar to a viral capsid. This microbial compartment is able to selectively draw ferrous iron (Fe^2+^) inside itself, where it is oxidized to form a mineral core of ferric oxy-hydroxide [14], a process that can later be reversed if iron becomes limiting. In the paper in this issue from Justin Bradley (@JustinB11698455), Joshua Fair, Andrew Hemmings and Nick Le Brun (@Nick_Le_Brun) from the University of East Anglia, UK, the authors look to understand how Fe^2+^ gets into a novel cyanobacterial ferritin. This protein has some unique features in its structure and mechanism that more closely resemble animal ferritins rather than other characterized bacterial ferritins, specifically in the active site of the ferroxidase [15]. Having checked my geometry, a rhombic dodecahedron is a hybrid of a cube and an octagon and hence the vertices are formed with both three or four edges meeting. For the cyanobacterial ferritin the iron entry point was thought to be at the threefold vertex [16] and here they test this idea by identifying two amino acids that are important for this function; one, an aspartate, is particularly important in coupling the entry of Fe^2+^ to the subsequent ferroxidase activity that forms the mineral. Altered ferritins that contain subunits with this mutant are less efficient at catalyzing mineral formation and also less able to release the Fe^3+^ from the microcompartment. This cyanobacterial ferritin then has a difference in mechanism to other bacterial ferritins and the authors point out that, unlike animals, bacteria have much greater diversity of ferritins, perhaps suggesting that their function in different environments where the rates of iron uptake and release could be quite variable are evolutionary advantageous.

Our final paper of this month is a Short Communication featuring a double-header of authors mentioned previously in *Musings*, with another paper from the group of Ruth Massey (@ProfRuthMassey) at the University of Bristol, UK, this time in collaboration with Andrew Edwards (@bugsinblood) from Imperial College, London. Following on from the paper I featured last month [17, 18], Dina Altwiley (@altwiley_dina) and colleagues, including Mario Recker (@MarioRecker), have been making further use of their large collection of bacteraemia-isolated strains of *Staphylococcus aureus* collected from hospitals in the UK and Ireland [19]. Here they are looking at capsule production across their collection, which they find is rather variable, as has been reported recently by others [20, 21], and likely explains why capsule-based vaccines have not been successful against *S. aureus* infections [22]. For the strains that lack capsule they look for single-nucleotide polymorphisms (SNPs) that correlate with this in their set of 300 strains and find them in the gene *agrC*, that encodes a known regulator of capsule production, but also identify other SNPs linked to this phenotype, including some in the *menD* gene, which is part of the menadione pathway required for the functioning of respiration. Mutations in *menD* also leads to the small colony variant (SCVs) phenotype [23, 24], and so they pose the question, does the SCV phenotype correlate with loss of capsule? By comparing strains with *menD* mutations with strains containing another SVC-forming allele in the *hemB* gene, they show a specific relationship between *menD* and capsule production not seen with the *hemB* alleles, suggesting that the relationship between SCVs and capsule production depends on the initial mutation causing the SCV phenotype.

As I close this month, just a reminder to look out for information about the Microbiology Society Annual Conference in Belfast 2022 and start thinking about offering a paper and how you could get involved with our celebrations for 75 years of the journal – omicron permitting, of course.
